# Artificial Intelligence-Driven Integration of ECG and Molecular Biomarkers in Pulmonary Embolism

**DOI:** 10.3390/ijms27020813

**Published:** 2026-01-14

**Authors:** Bojana Uzelac, Sanja Stanković

**Affiliations:** 1 Emergency Center, University Clinical Center of Serbia, Pasterova 2, 11000 Belgrade, Serbia; 2 Center for Medical Biochemistry, University Clinical Center of Serbia, Pasterova 2, 11000 Belgrade, Serbia; 3 Department of Biochemistry, Faculty of Medical Sciences University of Kragujevac, Svetozara Markovića 69, 34000 Kragujevac, Serbia

**Keywords:** pulmonary embolism, electrocardiogram, biomarkers, artificial intelligence

## Abstract

Pulmonary embolism (PE) is a serious cardiovascular condition and the third leading cause of cardiovascular mortality worldwide. However, its clinical presentation is often non-specific, making timely detection challenging. Biomarkers are commonly used to support early diagnosis and risk stratification. Molecular biomarkers provide information related to coagulation, inflammation, and cardiac injury. Electrocardiography (ECG) reflects cardiac functional changes caused by right ventricular (RV) stress and dilation secondary to increased pulmonary vascular resistance. Individually, these biomarkers have limited diagnostic accuracy. A promising approach to improving PE management involves integrating multimodal clinical data using Artificial Intelligence (AI). AI-based models can detect subtle patterns in ECG signals and molecular biomarker profiles that may be missed by conventional analysis. Combining these data sources may enhance diagnostic accuracy, refine risk assessment, and support personalized treatment. Despite ongoing challenges, including data quality, interpretability, and ethical considerations, AI-driven integration of ECG and molecular biomarkers represents a significant step forward in PE diagnosis and management. Further validation in large, prospective clinical studies is required.

## 1. Introduction

Pulmonary embolism (PE) is a cardiovascular condition characterized by the obstruction of one or more branches of the pulmonary arteries, usually by a thrombus from the deep veins of the lower extremities. This obstruction increases pulmonary vascular resistance, potentially leading to acute RV dysfunction, hypoxemia, hemodynamic instability, and, in severe cases, death [1].

PE is the third leading cause of cardiovascular mortality worldwide, which highlights the urgent need for timely diagnosis and appropriate management [2]. However, early detection is challenging due to the heterogeneity of clinical presentations, ranging from mild symptoms to sudden cardiovascular collapse.

According to the 2019 European Society of Cardiology (ESC) guidelines, accurate PE diagnosis requires a structured approach combining clinical assessment, imaging, and laboratory tests. For risk stratification, validated scores such as the Pulmonary Embolism Severity Index (PESI) and its simplified version (sPESI) help identify low-risk patients and highlight those who are hemodynamically unstable and at high risk of early mortality [1].

Biomarkers play an important role in the diagnostic and prognostic algorithm, complementing clinical scores and imaging. Molecular biomarkers reflect key pathophysiological processes, such as coagulation activation, systemic inflammation, and myocardial injury. For example, D-dimer is highly sensitive for ruling out PE in patients with low or intermediate clinical probability. Heart injury markers, such as troponin, and indicators of right ventricular strain, like BNP or NT-proBNP, help further refine risk, particularly in intermediate-risk patients [1]. Meanwhile, ECG provides additional information on functional changes, including RV dysfunction due to acute increases in pulmonary vascular resistance [3]. While each parameter offers valuable insights, their diagnostic and prognostic accuracy is limited when used alone, highlighting the need for integrative approaches.

Artificial intelligence (AI) has emerged as a promising tool to support PE management. By analyzing multimodal data—including ECG signals, molecular biomarker profiles, and clinical records—AI can identify subtle patterns that may be missed by conventional assessment. This approach maximizes the value of existing diagnostic information, enhances risk stratification, and supports more precise and consistent clinical decision-making [4]. Despite ongoing challenges related to data quality, interpretability, and ethical considerations, AI-driven integration represents a meaningful step forward in PE care. Further research, including large randomized clinical studies, is still needed to fully validate its clinical impact.

This article reviews the role of AI in integrating ECG and molecular biomarkers for PE management. We discuss the pathophysiological basis of PE, the individual strengths and limitations of ECG and molecular biomarkers, and examine how AI can help to maximize the information obtained from a multimodal approach.

## 2. Methodology

A selective literature search was conducted in the PubMed database using the key terms “pulmonary embolism,” “biomarkers,” “ECG,” and “artificial intelligence.”

## 3. Pulmonary Embolism: Clinical and Pathophysiological Background

### 3.1. Epidemiology and Clinical Significance of Pulmonary Embolism

Venous thromboembolism (VTE) represents the third most common acute cardiovascular condition, following myocardial infarction and stroke. The annual incidence of pulmonary embolism ranges from 39 to 115 per 100,000 population [2,5]. According to data from the United States, the incidence of PE in the general population is approximately 1 per 1000 individuals, corresponding to about 250,000 new cases annually [6]. The frequency of pulmonary embolism increases markedly with age, doubling with each decade after the age of 40 [7]. Data from the PIOPED study indicate an overall one-year mortality rate of 24% among patients with PE, while the cumulative recurrence rate during 10 years of follow-up reaches 30% [8]. Pulmonary embolism causes up to 300,000 deaths each year in the United States, and in six European countries with a combined population of 454.4 million, an estimated 370,000 deaths were attributed to VTE in 2004 [2,9].

Analysis of World Health Organization (WHO) mortality data from 73 countries between 2001 and 2023 revealed a global decline in the age-standardized mortality rate from pulmonary embolism, from 3.49 to 2.42 per 100,000. However, significant geographical and economic disparities remain: reductions were mainly seen in high-income countries, while lower-middle-income countries experienced increasing PE-related mortality [10].

The clinical severity of PE varies, with different proportions of patients presenting with mild, intermediate, or high-risk forms of the disease. High-risk pulmonary embolism, defined by hemodynamic instability, accounts for approximately 3–5% of all cases and is associated with early mortality exceeding 25%. Intermediate-risk PE comprises about 30–40% of patients with an elevated sPESI score (≥1), subdivided into intermediate–high risk, when both RV dysfunction and elevated biomarkers are present, and intermediate–low risk, when only one of these criteria is met. Low-risk PE represents the majority of cases (≈55–60%) and is characterized by hemodynamic stability, preserved right ventricular function, normal biomarkers, and a short-term mortality below 2% [1].

Early risk stratification of patients with pulmonary embolism is necessary, as therapeutic strategies differ substantially according to risk category. High-risk patients require urgent reperfusion therapy, including systemic thrombolysis or, in some cases, surgical intervention. Intermediate-risk patients are managed with anticoagulation alongside careful clinical and hemodynamic monitoring. Low-risk patients can be safely treated with anticoagulation alone, either in the hospital or on an outpatient basis, depending on their clinical condition.

### 3.2. Pathophysiology and Molecular Mechanisms Underlying Pulmonary Embolism

The cascade of pathophysiological responses at the molecular level in PE begins with obstruction of the pulmonary vasculature, which leads to endothelial injury and the release of endothelin-1 (ET-1), nitric oxide (NO), and von Willebrand factor. Damaged cells also release so-called Damage-Associated Molecular Patterns (DAMPs)—endogenous molecules that enhance the expression of adhesion molecules on endothelial cell surfaces (ICAM-1, VCAM-1, P-selectin) [11]. These effects are further enhanced by cytokines such as interleukin-8 (IL-8), which binds to its receptors CXCR1 and CXCR2 on neutrophils, inducing their chemotaxis toward the site of inflammation and activation [12].

Activated monocytes and macrophages form a multiprotein complex—the NLR family pyrin domain-containing-3 (NLRP3) inflammasome—which activates the enzyme caspase-1 and leads to the release of the biologically active form of interleukin-1β (IL-1β). (Through a HIF-1α-mediated mechanism, hypoxia has also been shown to be an essential factor in inflammasome activation.) The release of IL-1β triggers a series of pro-inflammatory signals that recruit and activate leukocytes, induce the production of additional cytokines and chemokines, and contribute to the formation of a pro-coagulant environment [13,14]. Inflammasome activation also induces the release of tissue factor (TF) from monocytes and macrophages, thereby initiating the extrinsic coagulation cascade and generating neutrophil extracellular traps (NETs) [15,16]. Recent studies also indicate the importance of IL-33 and its ST2 receptors in pulmonary inflammation in PE, through activation of the NF-κB (nuclear factor-κB) and MAPK (mitogen-activated protein kinase) pathways [17].

One of the most important pro-inflammatory cytokines in PE is IL-6, which stimulates TF production, induces fibrinogen synthesis, and increases plasminogen activator inhibitor-1 (PAI-1) expression, thereby reducing fibrinolysis. Also, IL-6 contributes to endothelial dysfunction, increases endothelial permeability, and enhances leukocyte and platelet adhesion [18].

In addition to the above mechanisms of immunothrombosis, oxidative stress with increased production of reactive oxygen species (ROS) also plays a vital role in the pathophysiology of PE. NOX enzymes (NADPH oxidases) play a key role. NOX2 generates superoxide anion (O2•−) by transferring electrons from NADPH to molecular oxygen, thereby initiating the formation of other ROS. Under PE conditions, this leads to disruption of vascular homeostasis, reduced NO bioavailability, and increased platelet aggregation. Moreover, superoxide anion and hydrogen peroxide (H_2_O_2_) often activate signaling pathways that lead to inflammatory responses and coagulation. At the same time, hydroxyl radical (•OH) and peroxynitrite (ONOO−) directly damage the endothelium and increase vascular permeability [19].

In conclusion, endothelial dysfunction and the release of vasoactive mediators lead to vasoconstriction and increased pulmonary vascular resistance, contributing to acute right ventricular strain. PE triggers a strong local and systemic inflammatory response, with activation of numerous cytokines and chemokines. These mediators further exacerbate pulmonary vasoconstriction, endothelial dysfunction, and changes in myocardial electrophysiology. At the same time, they activate both the extrinsic and intrinsic pathways of the coagulation cascade, resulting in greater thrombotic burden, which correlates with right ventricular dysfunction.

## 4. ECG as a Functional Assessment in Pulmonary Embolism

The ECG records the heart’s electrical activity, providing real-time information on conduction, rhythm, and myocardial stress. In PE, obstruction of the pulmonary arteries increases RV load, worsened by impaired gas exchange and redistribution of pulmonary blood flow. Acute RV overload, dilation, dysfunction, and possible ischemia, along with hypoxia, explain most ECG changes seen in PE patients [3,20,21,22].

The incidence of the most common ECG findings in PE is shown in Table 1.

One of the main advantages of ECG as a functional assessment is its universal availability. ECG machines are present in nearly all healthcare settings, including emergency departments, hospital wards, outpatient clinics, and ambulances. ECG recording is rapid, inexpensive, and non-invasive, making it particularly valuable in acute care settings. Moreover, ECG changes in PE have been shown to reflect disease severity and support risk stratification (Table 2).

Nevertheless, compared to the well-established roles of imaging and molecular biomarkers, the role of ECG in PE management remains limited and not fully defined. While numerous studies have shown correlations between specific ECG changes, right ventricular dysfunction, and worse outcomes [27,32,35,37,38], ECG has not been recognized as an independent prognostic functional assessment in official guidelines.

This limitation is largely due to the clinical characteristics of ECG. Some patients with confirmed PE may present with normal ECG findings, particularly in cases of small emboli. Moreover, many ECG abnormalities are nonspecific and can be observed in other conditions, such as chronic lung disease, myocardial ischemia, or right heart enlargement from different causes. For these reasons, ECG is currently used as a complementary functional assessment, offering information on cardiac stress rather than serving as a definitive diagnostic tool.

## 5. Molecular Biomarkers in Pulmonary Embolism

The term biomarker is defined as a measurable indicator of a normal or pathological biological process or of a physiological response to a therapeutic intervention [39]. In the context of pulmonary embolism, biomarkers play a key role in diagnosis, risk stratification, and outcome prediction, as emphasized in the official ESC guidelines.

### 5.1. D-Dimer

D-dimer represents the final product of fibrin degradation and reflects activation of the coagulation system and fibrinolysis, making it the most commonly used molecular biomarker in pulmonary embolism (PE). However, in diagnostic algorithms, its role is primarily limited to the exclusion of PE, as it exhibits high sensitivity (>95%) but low specificity (50–70%). In patients with low or intermediate clinical probability, normal D-dimer levels practically exclude PE [40]. Nevertheless, elevated levels are not diagnostic, as similar levels are seen in pregnancy, malignancy, infections, post-surgical states, or trauma [41]. Also, the ESC guidelines recommend using age-adjusted D-dimer cut-off values (age × 10 mg/L in patients > 50 years), as D-dimer levels naturally increase with age [1].

### 5.2. Troponin

Elevated levels of cardiac Troponin in PE reflect acute RV overload and ischemia caused by a sudden increase in pulmonary vascular resistance. Their prognostic significance in PE is well established [42]. A meta-analysis of 60 studies including 25,282 patients demonstrated a strong association between Troponin levels and in-hospital and 30-day mortality, right ventricular dysfunction, hemodynamic instability, and ICU admission. This association is consistent regardless of whether conventional (cTnI, cTnT) or high-sensitivity (hs-cTnI, hs-cTnT) assays are used [43,44]. While high-sensitivity Troponin can detect even minimal myocardial injury, it may overestimate clinical risk [45]. The prognostic role of Troponin is particularly important in hemodynamically stable patients with intermediate risk, where elevated levels identify individuals at increased risk of complications and early mortality [46]. Age-adjusted troponin cut-offs (TnT: 14 pg/mL for patients < 75 years, 45 pg/mL for patients > 75 years) further improve the negative predictive value [47]. Despite their prognostic utility, Troponins lack specificity for PE, as elevated levels can occur in other conditions such as acute coronary syndrome, heart failure, myocarditis, chronic kidney disease, and sepsis. Also, normal Troponin values do not exclude PE [48].

The primary value of troponin as a molecular biomarker in PE lies in its combination with natriuretic peptides, clinical scoring systems (PESI, sPESI), echocardiographic indicators of RV dysfunction, and findings from computed tomography pulmonary angiography (CTPA) [46,49]. This multimodal approach allows for more precise identification of intermediate–high-risk patients who require closer monitoring, potential reperfusion therapy, and treatment in an intensive care setting [40].

### 5.3. Natriuretic Peptides

Natriuretic peptides, including BNP and NT-proBNP, are released in response to RV pressure and volume overload, serving as markers of RV dysfunction. Similar to troponins, they hold significant prognostic value, particularly in intermediate-risk patients, where elevated levels are associated with higher complication rates and mortality [1]. However, their specificity remains limited, as elevated levels may also be observed in older individuals and in patients with heart failure, atrial fibrillation, or chronic kidney disease [41]. Conversely, low BNP or NT-proBNP levels reliably exclude early adverse outcomes, demonstrating high sensitivity and negative predictive value [49,50].

### 5.4. Inflammatory Markers

Inflammatory markers such as CRP, leukocytes, and hemogram-derived indices can be elevated in PE but lack diagnostic specificity. They serve as adjunctive biomarkers reflecting the systemic inflammatory response and may complement existing diagnostic algorithms, though their specificity remains limited and results often vary depending on measurement methodology and clinical context [51,52,53].

### 5.5. Limitations of Single Biomarker Approaches

Despite advances in understanding PE pathophysiology, significant challenges in diagnosis and risk stratification remain, largely due to the lack of highly specific and reliable biomarkers. Although molecular biomarkers provide valuable information on thrombotic burden, hemodynamic stress, ischemia, and RV dysfunction, none can independently confirm or exclude the diagnosis. Their utility is further constrained by the fact that they provide only a static “snapshot” of biochemical processes and do not directly reflect real-time changes in cardiac function. This limitation underscores the need for complementary physiological data, such as ECG parameters.

Also, relying on single biomarkers may lead to a false sense of security or unnecessary diagnostic and interventional procedures. Therefore, clinical guidelines emphasize combining biomarker assessment with clinical evaluation and imaging to support more accurate and reliable decision-making [1]. In our recently published study, we demonstrated that the Daniel ECG score and dNLR are independent predictors of PE severity, highlighting the significant value of a combined approach integrating ECG and molecular biomarkers [53]. Earlier, the ZATPOL-2 Registry study by Kukla et al. [54] showed that ECG signs of RV strain are strongly associated with elevated cardiac biomarkers (troponin and NT-proBNP) and echocardiographic evidence of RV dysfunction. Jiao et al. found that in normotensive PE, a simple multivariable model combining low QRS voltage on ECG, D-dimer, troponin, and PaO_2_/FiO_2_ predicted in-hospital adverse events with an AUROC of 0.85, outperforming the PESI, Bova, and FAST scores [55]. Also, echocardiography and biomarkers provide a more refined risk stratification in patients with normotensive PE, particularly within PESI low-risk classes [56].

Given that RV dysfunction is the central prognostic determinant in pulmonary embolism, biomarker data should be interpreted primarily with this in mind [57]. Future PE management algorithms are likely to focus on a multimodal approach, integrating several biomarkers and diagnostic tools rather than relying on a single universal marker.

## 6. Artificial Intelligence in Pulmonary Embolism Assessment

### 6.1. Introduction to Artificial Intelligence

Artificial intelligence (AI) refers to computer systems capable of performing tasks that typically require human intelligence, including learning, reasoning, pattern recognition, and decision-making. Unlike conventional programs based on fixed instructions, AI can analyze large and complex datasets, uncover intricate non-linear relationships, and adapt its conclusions based on new information. Recently, AI platforms have become some of the fastest-growing technologies across various fields, including medicine [58].

Medical practice, which relies solely on human resources, is often limited in terms of optimization, efficiency, consistency, and error reduction. AI offers significant potential to enhance these aspects through its applications in diagnostics, risk prediction, medical imaging and ECG interpretation, and the analysis of clinical parameters. The application of AI in cardiology is particularly promising, with significant results already reported in the analysis of pericardial effusions, long QT syndrome (LQTS), atrial fibrillation, hypertrophic cardiomyopathy (HCM) and the assessment of cardiac contractile dysfunction [59,60,61,62,63,64].

### 6.2. Application of AI in Pulmonary Embolism

Management of pulmonary embolism continues to lag behind that of other major cardiovascular conditions, such as myocardial infarction and stroke. This disparity in evidence and generally less favorable outcomes stems largely from clinical and organizational challenges, as highlighted in the introduction. Artificial intelligence presents promising opportunities to help overcome many of these limitations.

First, AI-based models enable more efficient integration of large-scale and heterogeneous data from electronic medical records, allowing faster and more accurate estimation of PE probability. The study by Sezer et al. [65] demonstrated that an AI model based on a multilayer perceptron (MLP) artificial neural network can predict the risk of PE using only clinical data, laboratory parameters, and clinical probability scores. The model achieved high diagnostic accuracy (96%) and specificity (89%), highlighting its potential value for early diagnosis and clinical decision support even before referral for CTPA. Similarly, the PERFORM study developed a machine learning (ML) model for PE risk prediction using longitudinal electronic health record data. This model outperformed traditional clinical prediction scores, such as the Wells and revised Geneva scores, particularly in outpatient settings [66]. Even better results were demonstrated in a study that used an XGBoost machine learning model based on routine laboratory and clinical data. This AI algorithm accurately predicts pulmonary embolism with a sensitivity and specificity of 99% and an AUROC of 0.992. This enables rapid assessment of PE probability and rational referral for CTPA [67].

More importantly, AI algorithms using deep learning architectures, such as nnU-Net, have shown reliable performance in directly detecting pulmonary embolism on CTPA, quantifying thrombus volume, and automatically calculating the right-to-left ventricular diameter ratio (RV/LV) [68]. The use of deep learning enables automated assessment of thrombotic burden, which shows strong correlation with established CTPA-based severity scores, such as Qanadli and Mastora [69].

Weikert et al. reported high diagnostic accuracy of an AI algorithm for automated PE detection on CTPA, achieving a sensitivity of 92.7% and a specificity of 95.5% [70]. In addition, Batra et al. demonstrated that AI-assisted worklist reprioritization significantly reduced radiologists’ report turnaround times for CTPA examinations positive for PE [71].

Furthermore, a meta-analysis by Soffer et al., which included seven studies and a total of 36,847 CTPA examinations, reported promising results for AI-based detection of acute PE. Deep learning algorithms, particularly convolutional neural networks (CNNs), showed high accuracy in identifying and localizing pulmonary emboli [4].

A systematic review comprising 12 studies with a total of 341,112 CTPA scans demonstrated the efficacy of AI algorithms, particularly convolutional neural networks (CNN), in the detection of pulmonary embolism. The algorithms showed high sensitivity (up to 90%) with a relatively low number of false-positive findings [72]. Some of these AI solutions have been implemented as mobile applications, facilitating rapid communication and coordination among clinicians involved in PE management. AI integration of early and accurate diagnosis, rapid clinician notification, and improved interdisciplinary coordination has the potential to significantly shorten the time to initiation of treatment in patients with PE [73,74].

In PE management, risk stratification represents one of the most challenging aspects of care. Clinical interpretation of multiple molecular biomarkers—each limited when considered alone—combined with predictive scores and criteria often results in substantial variability in treatment strategies. In contrast, AI-based machine learning approaches allow for more effective integration of complex and heterogeneous data, including treatment modalities, complications, and clinical outcomes. A large systematic review and meta-analysis encompassing 17 studies and 844,071 patients demonstrated that ML models outperform traditional risk stratification tools, such as PESI and sPESI, in predicting 30-day mortality in PE. The pooled sensitivity was 0.88, specificity 0.79, and AUROC 0.91, indicating excellent discriminative performance [75].

Finally, AI may play a crucial role in the continuous collection and analysis of PE-related data, reducing variability in management and promoting treatment standardization. The creation of large-scale clinical and imaging databases could support the development of new evidence-based protocols and future guidelines for PE management, further advancing personalized and standardized care.

## 7. AI-Based Analysis of ECG in Pulmonary Embolism

Although the ECG has long been used as a functional assessment in pulmonary embolism, its diagnostic and prognostic value has remained limited, both with regard to individual ECG findings and ECG-based scoring systems. The emergence of AI has shifted this perspective, raising the possibility that additional clinically relevant ECG features in PE exist but are not routinely recognized or are overlooked by clinicians. This led to a growing body of studies focused on AI-driven interpretation of ECGs in patients with PE, primarily based on the analysis of raw ECG signals and associated data.

In the study by Wysokinski et al. [76], a deep neural network-based AI (AI-DNN) analyzed 12-lead ECGs of patients diagnosed with PE by CTPA. For predicting all acute PE, the model showed only moderate performance; however, for PE with acute right ventricular strain or saddle PE, results were substantially better (AUROC 0.84; sensitivity 80.8%, specificity 64.7%). With high negative predictive values for any acute PE (NPV 94.54%) and especially severe PE (NPV 99.52%), this AI-driven ECG model has the potential to rapidly and reliably rule out PE.

Valente Silva et al. [77] developed an AI model based on an ensemble of neural networks using 12-lead ECGs for PE prediction. This algorithm demonstrated higher specificity than clinical scores recommended in guidelines (Wells and Geneva Scores combined with standard D-dimer cut-off of 500 ng/mL), as well as PEGeD and YEARS algorithms, showing it can confidently rule out PE in the studied population (AUROC 0.75; specificity 100%, sensitivity 50%).

The superiority of AI-based ECG models in predicting clinical PE classification was shown by Gokhale et al. [78]. Raw ECG signals were first filtered, followed by extraction of 419 spatiotemporal features encompassing morphology, amplitudes, and temporal relationships of electrical potentials. The AI-ECG model accurately identified patients with severe PE (AUROC 0.84), outperforming the S1Q3T3 score (AUROC 0.66) and Daniel score (AUROC 0.69).

Somani et al. [79] developed a machine learning algorithm to improve specificity in acute PE screening. Their Fusion model, combining ECG signals and clinical data, showed superior discrimination of PE (AUROC 0.84) compared with widely used clinical scores: Wells’ Criteria, Revised Geneva Score, Pulmonary Embolism Rule-Out Criteria, and 4-Level Pulmonary Embolism Clinical Probability Score (AUROC 0.50–0.58). This study also highlighted the advantage of AI in processing large clinical datasets and routinely collected ECG waveforms.

Another study evaluated a smartphone app that analyzes printed ECG recordings to generate digital biomarkers for various conditions, including RV dysfunction (QCG-RVDys) in acute PTE. The app demonstrated high reliability in excluding RV dysfunction (NPV 95.5%), high sensitivity (91%), and outperformed expert analysis, Troponin, and ProBNP [73].

Some AI algorithms based on ensemble neural networks have used ECG signals and demographic data for the early prediction of pulmonary hypertension (PH)—a key consequence and risk factor in PTE. In a study involving more than 70,000 ECG recordings, AI models achieved high predictive accuracy for PH (AUROC 0.86–0.90) [80]. Similar results were reported in a Mayo Clinic study, where an AI algorithm for early PH detection (PH-EDA) based on standard 12-lead ECG achieved AUROC values of 0.88–0.92 [81].

A large Canadian study analyzed over 1.6 million ECG recordings from 244,077 patients across 84 hospitals, developing deep learning models for 15 cardiovascular diagnoses, including PE. However, the DL model using only ECG, age, and sex achieved the lowest performance for PE (AUROC 68.9%), indicating only moderate diagnostic ability compared with, for example, STEMI (AUROC 95.5%) [58].

Marques et al. [82] tested different neural network architectures and showed that transfer learning (leveraging knowledge from larger general ECG datasets such as PTB-XL, CPSC18, and MedalCare-XL) significantly improved performance on a smaller, PE-specific dataset. While models can detect subtle ECG signals associated with PE, current evidence indicates that ECG alone is not yet a reliable standalone diagnostic tool without integration with other clinical information.

## 8. Integrating ECG and Molecular Biomarkers Using AI

The AI-Mayo PE (AIM PE) study investigated the integration of ECG and biomarkers, particularly D-dimer, in an AI algorithm for predicting pulmonary embolism. When used alone, the AI-ECG model had an AUROC of 0.69, with the ability to stratify patients into low, intermediate, and high-risk categories. The addition of a negative D-dimer in the intermediate-risk group increased the negative predictive value to over 99.9%, significantly reducing the need for CTPA imaging. The combined AI-ECG + D-dimer approach using a machine learning algorithm (AdaBoost) achieved an AUROC of 0.93, substantially outperforming classical logistic regression (AUROC 0.79) [83].

The study by Villacorta and colleagues [84] demonstrated that the combination of clinical variables and D-dimer significantly contributes to machine learning models for predicting PE. Including D-dimer increased the AUROC from 0.73 to 0.89, surpassing traditional scores such as Wells, revised Geneva, and PERC. The model also enabled better exclusion of PE with a low rate of false-negative results compared to alternative approaches.

By combining clinical variables with biomarkers such as D-dimer and cardiac troponin T, machine learning can support rapid and accurate PE diagnosis. In the study by Xi and colleagues, the Random Forest algorithm achieved the highest diagnostic performance (AUROC 0.774) and outperformed the Wells score, revised Geneva score, and YEARS algorithm [85].

However, very few studies have systematically examined integrated models combining ECG and molecular biomarkers, highlighting the need for further research to confirm their clinical utility.

## 9. Clinical Implications and Benefits of Artificial Intelligence in Pulmonary Embolism

Recently, artificial intelligence has emerged as a potential “missing link,” capable of addressing many of the challenges encountered throughout pulmonary embolism management.

AI-driven algorithms enable earlier disease recognition, improvement of diagnostic procedures, and better coordination within the clinical team. This is achieved through multimodal analysis of electrocardiographic patterns, molecular biomarkers, and clinical data derived from medical records. As a result, AI enables early identification of patients with a high likelihood of PE who require further diagnostic evaluation, particularly CTPA. This more precise selection helps reserve urgent imaging for patients who truly need it, while optimizing resources and reducing radiation exposure. In addition, during the interpretation of CTPA findings, AI models may detect subtle radiological changes that often remain unnoticed in routine clinical assessment, significantly shortening the overall time to PE diagnosis [86,87].

Risk stratification and prognostic assessment remain among the weakest aspects of current PE management [87]. Integrating AI into clinical practice could help identify high-risk patients and improve outcome prediction, allowing for a more individualized treatment approach.

AI may be particularly valuable in emergency medicine, where clinicians often face system overload, staff shortages, and the need for rapid decision-making [71]. Support in triage and patient reprioritization could provide meaningful assistance in everyday clinical practice.

In summary, the application of artificial intelligence in PE management can enable faster and more accurate risk assessment and support improved therapeutic planning. It can also promote more efficient use of healthcare resources, with the potential to enhance clinical outcomes and patient safety.

## 10. Limitations, Challenges and Ethical Considerations

However, before AI can be widely implemented in clinical practice, certain questions and challenges must be addressed.

First, there is the issue of data quality and availability. Artificial intelligence models rely on large volumes of high-quality data, and the lack of standardized and representative datasets can affect the accuracy and reliability of predictions.

The next challenge is generalizability and validation. Many AI models are trained on limited, specific datasets, making it difficult to apply their results across different healthcare systems or demographic groups. Therefore, independent external validation using separate datasets is essential before broader clinical adoption. Without it, there is a risk that AI tools may perform well in controlled research settings but poorly in real-world clinical practice.

Integration into clinical workflows also remains a challenge. Implementing AI within hospital protocols requires staff training and adaptation of existing care algorithms. Without careful system design, “alert fatigue” may occur, where clinicians begin to ignore or overlook warnings, reducing effectiveness and compromising patient safety. Preventing this requires interdisciplinary collaboration among developers, clinicians, biostatisticians, and regulatory authorities.

Interpretability and trust represent further challenges. Deep neural networks, as well as other AI models, can be difficult to understand. Clinicians must comprehend the basis of predictions to maintain justified trust in the algorithm. Additionally, AI models require regular updating to reflect new clinical practices and demographic changes. Continuous performance monitoring is crucial to ensure safety and effectiveness.

Finally, but no less importantly, ethical and legal issues remain. Responsibility for errors remains uncertain, highlighting the need to clearly define accountability for AI-assisted diagnoses. Patient privacy and data security must also be safeguarded in accordance with regulations. Lastly, it is important to recognize that such high-tech support will not be equally available worldwide, which may exacerbate healthcare disparities [88].

## 11. Future Directions

First, it is important to recognize that AI should not replace the clinician but rather complement their expertise through collaborative use. This approach preserves clinical judgment, adherence to best-practice guidelines, empathy toward the patient, and ethical care.

In the future, advances in emerging scientific fields such as genomics, proteomics, and metabolomics are expected to generate novel biomarkers that can enhance AI models. Integrating these data with ECG and traditional biomarkers will allow for even more precise diagnostic and prognostic algorithms. Adequate ECG monitoring could enable longitudinal tracking of patient data, facilitating early recognition of right ventricular strain, a key prognostic determinant in PE.

The likely future of AI-driven multimodal PE management lies in personalized medicine, where risk assessments and therapeutic decisions are tailored to each patient’s individual profile [89].

Future research should focus on large, multicenter prospective studies with standardized data collection and sharing, transparent reporting of AI model performance, and clinical trials assessing impact on outcomes, efficiency, and cost.

## 12. Conclusions

Pulmonary embolism remains a significant diagnostic and prognostic challenge due to the heterogeneity of its clinical presentation and the lack of a specific diagnostic marker. Although ECG and molecular biomarkers provide valuable information, they are insufficient on their own to fully assess disease severity and risk. Artificial intelligence offers the potential to integrate these complementary data, uncover subtle patterns, and enable earlier and more accurate diagnosis, improved risk stratification, and a personalized approach to patient management.

Evidence for the use of AI in PE management is still emerging. Most studies focus on algorithm development rather than direct clinical outcomes, and the generalizability of models is limited by dataset size and diversity. Nevertheless, careful implementation of AI—supported by rigorous validation, ethical frameworks, and close collaboration with clinicians—has the potential to significantly advance the care of patients with PE.

In the future, AI-driven multimodal approaches could optimize emergency care and patient triage, as well as support the development of personalized medicine. This would enable tailored therapeutic strategies, more efficient use of resources, greater safety, and improved clinical outcomes.

## Figures and Tables

**Table 1:** Incidence of the most significant ECG findings in PE according to different studies.

Study (First Author, Year)	*n*	Most Significant ECG Findings (Incidence)
**Qaddoura, 2017 [23].**	9198	S1Q3T3 (20–50%), RBBB (6–20%), TWI in leads V1–V4 (22–68%), RAD (4–42%), AF (8–13%), STE-aVR (up to 36%)
**Shopp, 2015 [3].**	8209	Tachycardia (38%), TWI V1 (38%), STE in aVR (36%), S1Q3T3 (24%), RBBB (10%), AF (8.6%)
**Digby, 2015 [24].**	36 studies	S1Q3T3 (11–52%), RBBB (6–69%), TWI V1–V4 (16–68%), STE in aVR (30–43%), STE ≥ of 1 mm in any other lead (16–48%)
**Kukla, 2014 [25].**	500	S1Q3T3 (32.6%), qR/QR in V1 (11.3%), RBBB (12.6%), STE in III (12.5%), STE in V1 (23.6%), STE in aVR (36.2%), TWI in leads V2–V4 (40.3%)
**Geibel, 2005 [26].**	508	Sinus tachycardia (67% in survivors vs. 77% in non-survivors), RBBB/iRBBB (33% vs. 41%), Q waves in III/aVF 87.2% vs. 14%), STE in I, II, V4-V6 (5.8% vs. 16%), TWI V2–V3 (50% vs. 44%)
**Alarcon, 2019 [27].**	684	Sinus tachycardia (51.7%), S1Q3T3 (24.5%), TWI in leads V1–V4 (16%), RBBB (9.9%), AF (8.6%)
**Casazza, 2018 [28].**	1194	TWI in leads V1–V3 (28.4%), S1Q3T3 (24.4%), RBBB (22.4%), Qr in V1 (6.8%)
**Salimi, 2021 [29].**	733	TWI (41.9%), S1Q3T3 (38.6%), STE in aVR (28.2%), iRBBB (18.9%), STE in V1 (16.8%)
**Zuin, 2022 [30].**	687	RBBB (16.9% non-high risk vs. 45.1% high-risk), TWI in leads V1–V4 (27.6% vs. 37.4%), qR in V1 (5.4% vs. 12.7%)
**Bolt, 2019 [31].**	390	≥1 RV strain sign (82%), TWI in leads V1–V4 (49%), S1Q3T3 (15%), RBBB (15%)
**Wang, 2023 [32].**	341	S1Q3T3, ST (17.1%), RBBB/iRBBB (12.2%), RAD (2.4%), atrial arrhythmias (17.1%), TWI in leads V1–V3 (12.2%)
**Thomson, 2019 [33].**	189	Sinus tachycardia (28%), RV strain (11.1%), RBBB (9.0%), S1Q3T3 (3.7%), P pulmonale (0.5%)
**Vanni, 2009 [34].**	386	RV strain (34%), TWI in leads V1–V3 (16%), S1Q3T3 pattern (15%), RBBB (10%)
**Weekes, 2022 [35].**	1676	RBBB/iRBBB (no clinical deterioration 13.2% vs. 20.5% with clinical deterioration), S1Q3T3 pattern (14.4% vs. 22.4%), TWI in leads V2–V4 (11.88% vs. 20.5%), TWI in leads II, III, and aVF (8.6% vs. 14.7%), STE in lead V1 (7.9% vs. 12.8%)
**Bahreini, 2024 [36].**	250	Q wave in lead III (25.2%), TWI in V1–V3 (24.4%), TWI in V4–V6 (14.8%), S1Q3T3 pattern (7.6%), RBBB (3.2%)

RBBB—right bundle branch block; TWI—T-wave inversion; STD—ST-segment depression; RAD—right axis deviation; STE—ST-segment elevation; AF—atrial fibrillation.

**Table 2:** Pathophysiological mechanisms and clinical significance of ECG changes in PTE^3^.

ECG Changes	Pathophysiological Mechanisms	Clinical Significance
**Sinus tachycardia**	Stress, pain, hypoxia	Frequent but non-specific
**Atrial arrhythmias**	Hypoxia, hemodynamic overload	Higher mortality
**S1Q3T3 pattern**	Acute RV overload	Rare; non-specific
**TWI (V1–V4)**	Acute RV overload and RV dysfunction	RV strain; severe PE
**STD**	Myocardial injury	Rare; poor prognosis
**RAD**	Acute RV overload	Higher specificity, but rare
**Low voltages**	Reduced RV electrical potential and stress	Poor prognosis, but non-specific and relatively rare
**P pulmonale**	Right atrial overload	Rare, no prognostic value
**Pseudo-infarct pattern**	Acute RV overload and RV dysfunction	Higher mortality
**STE (aVR, V1)**	RV ischemia	Severe PE, poor prognosis

TWI—T-wave inversion; STD—ST-segment depression; RAD—right axis deviation; STE—ST-segment elevation.

## Data Availability

No new data were created or analyzed in this study. Data sharing is not applicable to this article.
